# Risk Factors Associated with Severity of Nongenetic Intellectual Disability (Mental Retardation) among Children Aged 2–18 Years Attending Kenyatta National Hospital

**DOI:** 10.1155/2018/6956703

**Published:** 2018-04-18

**Authors:** Mathieu Nemerimana, Margaret Njambi Chege, Eunice Ajode Odhiambo

**Affiliations:** ^1^School of Nursing Sciences, College of Health Sciences, University of Nairobi, P.O. Box 19676-00202, Nairobi, Kenya; ^2^Department of Nursing and Midwifery, Kibogora Polytechnic, P.O. Box 31, Rusizi, Rwanda

## Abstract

**Background:**

Many of the nongenetic causal risk factors of intellectual disability (ID) can be prevented if they are identified early. There is paucity on information regarding potential risk factors associated with this condition in Kenya. This study aimed to establish risk factors associated with severity of nongenetic intellectual disability (ID) among children presenting with this condition at Kenyatta National Hospital (KNH).

**Methods:**

A hospital-based cross-sectional study was conducted over the period between March and June 2017 in pediatric and child/youth mental health departments of Kenyatta National Hospital (KNH), Kenya. It included children aged 2–18 years diagnosed with ID without underlying known genetic cause.

**Results:**

Of 97 patients with nongenetic ID, 24% had mild ID, 40% moderate, 23% severe-profound, and 10% unspecified ID. The mean age of children was 5.6 (±3.6) years. Male children were predominant (62%). Three independent factors including “labor complications” [AOR = 9.45, 95% CI = 1.23–113.29, *P* = 0.036], “admission to neonatal intensive care unit” [AOR = 8.09, 95% CI = 2.11–31.07, *P* = 0.002], and “cerebral palsy” [AOR = 21.18, CI = 4.18–107.40, *P* ≤ 0.001] were significantly associated with increased risk of severe/profound nongenetic ID.

**Conclusion:**

The present study findings suggest that perinatal complications as well as postnatal insults are associated with increased risk of developing severe-profound intellectual disability, implying that this occurrence may be reduced with appropriate antenatal, perinatal, and neonatal healthcare interventions.

## 1. Introduction

Intellectual disability (ID), formerly known as “mental retardation,” is a condition characterized by significant below average intellectual functioning and impairment in adaptive behaviors, manifested before age 18 years. The degrees of intellectual disability include mild, moderate, and severe and profound intellectual disability [1, 2]. Intellectual disability is a public concern due to the number of people affected by this condition with consideration of the increased demand of specialized medical, psychosocial, and educational services required to improve their quality of life [3]. According to recent reviews and meta-analysis, globally, about 1% of general population is affected by intellectual disability [4, 5]. Child/adolescent population has higher prevalence (18.30/1000) than the adult population (4.94/1000) [5]. Furthermore, it is estimated that about 1% of children between the ages 3 and 10 years are affected by intellectual disability worldwide [6].

Intellectual disability is associated with multicausal risk factors including genetic and nongenetic or acquired causes. But in some cases, the aetiology is unknown [7]. Genetic factors such as chromosomal abnormalities, inherited genetic traits, and single gene disorders are the major causes accounting for 30% to 50% of all intellectual disability cases. Nongenetic causes comprise prenatal, perinatal, postnatal, and environmental factors [8]. Most prevalent reported nongenetic prenatal risk factors include maternal conditions such as asthma, diabetes, hypertension, renal conditions, and epilepsy [8, 9]. Other factors are tobacco or alcohol use, parental advanced age, low maternal education, multiparity, and maternal black race [8]. Main perinatal factors are low birth weight, preterm birth, birth complications, and perinatal infections [8, 10]. Postnatal infections, exposure to toxicants like lead or mercury, developmental disorders, central nervous system malignancies, and chronic severe malnutrition have been reported as postnatal factors [7, 8]. These associated factors of intellectual disability are in interactive complexity with environmental factors and sociodemographic and socioeconomic characteristics of population [9, 11]. It is that many of the factors and causes of nongenetic intellectual disability are preventable, if early detection is done and timely interventions are taken [6, 12].

Most of the studies regarding risk factors of intellectual disability have been conducted in developed countries, with limited information from developing countries [3]. In Kenya, information on incidence, prevalence, and associated factors of intellectual disability is scarce. Studies are needed to establish more information on the burden of this condition in Kenya. Furthermore, little is known on the magnitude of potential nongenetic risk factors contributing to development of intellectual disability among children affected with this condition in Kenya. Given the paucity of epidemiological information on the causal risk factors associated with intellectual disability in Kenya, the aim of this study was to explore potential risk factors associated with development of severe/profound nongenetic intellectual disability among children presenting with this condition at Kenyatta National Hospital (KNH).

## 2. Methods

### 2.1. Study Setting and Design

This study was a hospital-based, descriptive cross-sectional study conducted over period from March, 2017, to June, 2017, in the pediatric and mental health departments of Kenyatta National Hospital (KNH). KNH is the biggest national referral hospitals in Kenya located in Nairobi, capital city of Kenya. This hospital is located in Upper-Hill area along hospital road, off-Ngong' road, Nairobi. Its total bed capacity is 2000. KNH has 50 inpatient wards and different outpatient and specialized clinics, among them are pediatric department and mental health department. The mental health department provides different services, among them are child psychiatric clinic and youth mental health clinic, both working on outpatient basis. Patients with mental health problems requiring inpatient care were being admitted to the general pediatric-medical wards. At KNH, children with intellectual disability with age of up to 12 years are followed up at the child psychiatric clinic and at pediatric neurologic outpatient clinic while the adolescents aged from 13 years are followed up at the youth mental health clinic.

### 2.2. Study Participants

Children/adolescents between the ages of 2 and 18 years and diagnosed with intellectual disability without underlying known genetic cause were recruited consecutively from pediatric wards, pediatric outpatient clinics, and mental health department of KNH, over a period of 4 months. The age group (2 to 18 years) of children was chosen based on the fact that DSM-5 (Diagnostic and Statistical Manual of Mental Disorders, Fifth Edition) criteria specify that diagnosis of intellectual disability is made during development period, before age of 18 years [1]. Participants were selected after the confirmation of the diagnosis of intellectual disability by a pediatrician and clinical psychologist. Children who were identified with genetic conditions known to lead to intellectual disability were excluded from the study. Those who were critically ill were also excluded from the study. Using purposive sampling method, a total of 97 patients with nongenetic intellectual disability were consecutively recruited as study participants over the 4-month period of data collection.

### 2.3. Data Collection and Analysis

Data was collected from consenting parents and through desk reviews of patient files. The details on degree of intellectual disability were obtained from the patient's medical records. Using semistructured questionnaire, data on child and parental sociodemographic characteristics, pregnancy, birth history, postnatal history, medical and nutritional histories, and environmental exposure were collected from the child's parents. This was supplemented by data from desk reviews. Information on the comorbid conditions was also checked from the medical records.

Data was analyzed using Statistical Package for Social Sciences (SPSS) 23.0 version (IBM SPSS Statistics v23). The relationships between the individual factors and severity of intellectual disability were evaluated using crude odds ratio for bivariate and adjusted odds ratio for multivariate logistic regression models. Significance of statistical association was tested using confidence interval (CI) of 95% and *P* value < 0.05.

### 2.4. Ethical Consideration

The ethical approval and permission to conduct the study was granted by the Kenyatta National Hospital-University of Nairobi Ethics and Research Committee (KNH/UON-ERC) (Approval number: P961/12/2016). The permission to collect data was provided by the Kenyatta National Hospital (KNH) administration. Written parental permission/informed consent was obtained from the parents of children with intellectual disability attending KNH. Assent was also obtained from adolescent without profound intellectual disability.

## 3. Results

### 3.1. Sociodemographic Characteristics of the Children and Parents

Of 97 children/adolescents included in the study, the mean age was 5.6 years (SD ± 3.6 years), majority were males (61.9%), and most (74.2%) were living with both of their parents.  Table 1 provides details on the sociodemographic characteristics of the children.

Table 2 shows the distribution of selected sociodemographic and socioeconomic characteristics among the parents. Majority of children's parents (mothers (82.5%) and fathers (58.7%)) were in middle ages between 21 and 35 years. The highest percentage (47.4%) of mothers were casual workers and only 31% were regularly employed, while about 21.6% were unemployed. More than half of the fathers were having regular employment. Majority of the mothers (57.7%) and fathers (58.7%) had attained secondary school education. 29.3% of fathers were smokers. Majority 53.6% of parents reported earning between 21,000 and 50.000 Kenyan Shillings as their monthly family income.

### 3.2. Bivariate Analysis of Factors Associated with Severity of Nongenetic Intellectual Disability among Children

There was more proportion of severe/profound intellectual disability among children of single mothers (38.9%) compared to those children raised by both parents (23.4%). However, this was not statistically significant [OR = 2.08; 95% CI = 0.69–6.31; *P* = 0.196]. The study showed no significant association between the other sociodemographic characteristics of children and severity of intellectual disability (Table 3).

Table 4 shows the relationship between sociodemographic and economic characteristics of parents and severity of nongenetic intellectual disability. Even though mothers aged above 35 years had increased proportion of children with severe/profound nongenetic intellectual disability (46.7%) compared to those aged between 21 and 35 years (21.7%), this was not statistically significant [OR = 3.15; 95% CI = 0.98–10.09; *P* = 0.053]. Children of unemployed mothers and those of fathers with regular employment were having increased odds of severe/profound intellectual disability compared to others; however, this was not statistically significant [OR = 2; 95% CI = 0.56–7.09; *P* = 0.283 and OR = 2.60; 95% CI = 0.82–8.20; *P* = 0.096, resp.]. Similarly, there was no significant association between severity of nongenetic intellectual disability and other parental sociodemographic characteristics.

The relationship between pregnancy-related factors and severity of intellectual disability was analyzed. On the variable “took any drugs during pregnancy,” there is a significant increase in the number of children with mild/moderate intellectual disability in mothers who denied having taken drugs during pregnancy. In the case of the severe/profound intellectual disability, the number of cases was the same to the mothers who did not take drugs. There were more children with intellectual disability, born of mothers who had indicated “living in environment where people smoke” than those who reported otherwise, and this was significant [OR = 0.18; 95% CI: 0.06–0.55;* P* = 0.001] (Table 5).

Table 6 shows the relationship between birth history of the children and severity of intellectual disability. Children born through labor complications had significantly more proportion of severe/profound intellectual disability (39.1%) [OR = 5.46; 95% CI = 1.66–18.02; *P* = 0.003] compared to those children without labor complications (10.5%). There was significantly higher proportion of severe/profound intellectual disability among children delivered by caesarean section (50.0%) [OR = 4.64; 95% CI = 1.61–13.38; *P* = 0.005] than those children delivered through spontaneous vaginal delivery (17.7%) and this was statistically significant. Apgar score at birth was also significantly associated with severity of intellectual disability among children. Children with Apgar score lower than 7 out of ten significantly suffered severe/profound intellectual disability (38.6%) [OR = 4.41; 95% CI = 1.44–13.46; *P* = 0.007] more than children who scored above 7 out of ten (12.5%). Similarly children who were resuscitated at birth had significantly higher proportion of severe/profound intellectual disability (40.0%) [OR = 4.22; 95% CI = 1.45–12.29; *P* = 0.006] than those who were not (13.6%).

There was significantly increased proportion of severe/profound intellectual disability among children who had any neonatal difficulties (36.5%) [OR = 5.57; 95% CI = 1.49–20.75; *P* = 0.006] than those children without (9.4%). Children who were admitted to NICU during neonatal period had significantly more proportion of severe/profound intellectual disability (41.9%) [OR = 6.66; 95% CI = 2.01–22.03; *P* = 0.001] compared to those children that have never been admitted in NICU during neonatal period (9.8%). Similarly, those children with neonatal breathing difficulties (37.8%) had higher proposition of severe/profound intellectual disability than others without neonatal breathing difficulties (17.0%) and this was significant [OR = 2.97; 95% CI = 1.08–8.15; *P* = 0.031]. There was also significant association between neonatal feeding difficulties and severe/profound intellectual disability where children with neonatal feeding difficulties had significantly more severe/profound intellectual disability (50.0%) [OR = 3.86; 95% CI = 1.23–12.09; *P* = 0.016] compared to those children without (20.6%). No statistical significant relationship was found in the factors such as neonatal seizures, neonatal infection, and neonatal jaundice (Table 7). There was no statistically significant association observed between infant and childhood medical and severity of intellectual disability among the children (Table 8).

Table 9 shows the bivariate analysis of relationship between preexisting/comorbid and severity of intellectual disability. Children with cerebral palsy were significantly more likely to suffer severe/profound intellectual disability [OR = 18.18; 95% CI = 3.88–85.14; *P* ≤ 0.001] compared to those children without. There was no statistically significant association observed in other variables.

### 3.3. Multivariate Analysis of Factors Associated with Severity of Nongenetic Intellectual Disability among Children

Binary logistic regression analysis was applied to identify the variables independently associated with severity of intellectual disability among children aged 2 to 18 years. Eleven (11) factors were considered in the analysis including labor complications, mode of delivery, APGAR score at birth, whether the baby was resuscitated at birth, any neonatal difficulties, whether the baby was admitted in NICU, neonatal breathing difficulty, neonatal feeding difficulties, cerebral palsy, using drugs during pregnancy, and living in environment where people smoke. Upon fitting these factors using binary logistic regression and by specifying* “backward LR” *method with removal at *P* < 0.05, three (3) factors remained in the final analysis (Table 10). Severe/profound intellectual disability was about 10 times more among children with labor complications during birth [AOR = 9.45; 95% CI = 1.23–113.29; *P* = 0.036] compared to those children without labor complications. Children who were admitted to nursery during neonatal period had 8 times more likely to have severe/profound intellectual disability [AOR = 8.09; 95% CI = 2.11–31.07; *P* = 0.002] compared to those children that have been never admitted in nursery during neonate. Children with cerebral palsy were 21-fold more likely to have severe/profound intellectual disability [AOR = 21.18; 95% CI = 4.18–107.40; *P* ≤ 0.001] compared to those children without cerebral palsy.

## 4. Discussion

The study findings indicate that mean age of study population was 5.6 years with a standard deviation of 3.6 years. Male children were more affected than females; this finding is in agreement with the results from other studies which reported male predominance [4, 5, 13]. Current findings show that high proportion (40%) of children were suffering moderate intellectual disability. This result agrees with a similar study conducted in India [14] where 40% of children had moderate intellectual disability. This high proportion of moderate intellectual disability could be attributed to referrals as KNH serves as a referral hospital. Children with moderate intellectual disability tend to have remarkable limitations in meeting expected standards of personal independence and social responsibility in different aspects of daily life, especially when they start school. Therefore, when child starts to show slow academic achievements, he/she is referred for psychological evaluation. Logistic regressions analysis did not reveal any significant association of child sociodemographic variables (including age, gender, family set-up, and number of children in family) and severity of intellectual disability. This result is similar to the findings of a study done in India [14] which examined correlation of sociodemographic variables of patients with intellectual disability and types of intellectual disability.

In the present study, mothers aged 35 years and above were having increased proportion of children with severe-profound intellectual disability compared to those aged 21–35 years. However, there was no statistically significant relationship between parental age and severity of intellectual disability. An early study done by Drews et al. [15] and a recent systemic review and meta-analysis done by Huang et al. [8] reported a positive association between advanced parental age and intellectual disability though these studies were combining both genetic and nongenetic cases. The present study included only children with intellectual disability that is considered nongenetic, with exclusion of those who were having genetic disorders known to lead to intellectual disability. This fact may explain the predominance of parents with middle ages in this study. No relationship was revealed between parental level of education and severity of intellectual disability. However, a review and meta-analysis by Huang et al. reported positive association of lack of maternal education with intellectual disability [8]. Moreover, one of the findings from a study carried out in Utah, America, indicated a significant association between intellectual disability (with exclusion of genetic cases) and maternal education though that was not significant on paternal education [10].

Even though socioeconomic status of the parents did not show any statistically significant relationship with the severity of intellectual disability, great proportion of children with severe-profound intellectual disability were found among mothers who were unemployed compared to those with employment. Comparable findings were reported in Indian study in which no significant association of intellectual disability severity and socioeconomic status was found [14]. A cohort study done in Brazil reported also lack of association between socioeconomic status and groups with intellectual disability [16]. On the contrary, a study done in Australia found positive relationship of socioeconomic disadvantage and increased risk of intellectual disability [17]. Probably, this difference may be due to the fact that the latter studies used different methods and large population compared to the present study.

Tobacco smoking and use of alcohol during pregnancy was found to be a major risk factor for developing intellectual disability in the offspring [4, 8]. In this study, no association was found between alcohol use or tobacco smoking during pregnancy and severity of intellectual disability. O'Leary et al., in their population based study to examine the association of maternal alcohol use disorder and intellectual disability, found that children of mothers with an alcohol-related diagnosis recorded during pregnancy had three times increased risk of intellectual disability than others [18]. Unexpectedly, inverse association with severity of intellectual disability was found in mothers who reported living in the environment where people smoke.

In this study, no significant association was found between severity of intellectual disability and place of delivery or gestational age. Bivariate analysis demonstrated significant association of delivery through caesarean section and intellectual disability severity, though this lost its significance in multivariate analysis. This quietly corroborate results reported by Langridge et al. in which there was an increased risk of intellectual disability (ID) in children born via caesarean section compared to those delivered through a spontaneous vaginal birth [19] and the study by Bilder et al. which found significant association of primary/repeat caesarean sections and intellectual disability [10]. The present study did not establish what are the indications of caesarean sections. It is however difficult to conclude from this study whether merely caesarean section as mode of delivery is a risk factor for intellectual disability. It is therefore necessary to further examine the relationship between the various indications of caesarean section with the incidence of intellectual disability.

The children born through complicated labor had increased likelihood of severe/profound intellectual disability; they had about 10 times risk of severe/profound intellectual disability compared to others without history of labor complications. This compares with the findings from other studies that report positive and significant association of labor and delivery complications and degrees of intellectual disability [5, 19]. Labor and delivery complications lead to complications such fetal distress and birth asphyxia and intracranial hemorrhage leading to poor neonatal outcomes which later affect child brain development due to damage resulting from hypoxia related to these complications.

Low Apgar score is risk factor for intellectual disability [10]. In this study, bivariate analyses, children with histories of lower Apgar score, neonatal complications, and resuscitation at birth had increased risks of having severe-profound intellectual disability compared to their counterparts without these histories, though this association lost its significance after multivariate regressions analysis. The histories of perinatal difficulties and neonatal resuscitation required at birth were distinctly shown to be associated with increased in intellectual disability [19]. Low Apgar scores indicate poor birth outcomes with need of neonatal resuscitation, thus increasing probability of neonatal sequelae which expose the child to develop intellectual disability during development period. This is supported by evidence from cohort study conducted in Brazil where 13.2% of intellectual disability cases were attributed to neonatal sequelae [16].

Karam et al. noted that some of the neonatal complications and problems may cause neonatal sequelae resulting in intellectual disability [16]. In this study, children with history of neonatal breathing difficulty and those with neonatal feeding difficulties, respectively, had three and four odds of having severe-profound intellectual disability compared to their counterparts. However, these associations were not significant in multivariate logistic regressions. Children who were admitted to neonatal intensive care unit (NICU) were having 8 times risk of having severe-profound intellectual disability compared to their counterparts not admitted in NICU. This could be attributed to the fact that labor complications lead to birth difficulties and neonatal complications which increase the probability of being admitted in neonatal intensive care unit. Being born with birth complications suggests increased risk of debilitating conditions which predict likelihood to be affected with intellectual disability. Maulik et al. indicated neonatal infections among the common postnatal causes [5]. The present study did not find any significant association between severity of intellectual disability and medical histories of neonatal seizures, neonatal infection, and neonatal jaundice.

Children were reviewed for presence of any coexisting medical and mental-psychiatric comorbid conditions. Children with cerebral palsy were twenty-one-fold more likely to have severe-profound intellectual disability compared to others without it. This result is supported by early findings which also observed an increased risk of intellectual disability in children with cerebral palsy [20]. There was no statistical significant relationship found between severity of intellectual disability and other comorbid conditions.

## 5. Conclusions

Severity of intellectual disability is positively and significantly associated with environmental factors. Perinatal and postnatal insults including labor complications and being admitted to neonatal intensive care unit during neonatal period were significantly associated with increased risk of severe/profound intellectual disability. Children with cerebral palsy were at a more risk of having severe/profound intellectual disability than those without it.

## 6. Study Limitations

Being a hospital-based, descriptive cross-sectional study, this study included only study participants attending KNH; therefore findings may not reflect actual factors from general population in the country, and so results might not be generalizable. Because of the nature of study design, being a cross-sectional study, it limits its utility for causal inference.

## Figures and Tables

**Table 1:** Distribution sociodemographic characteristics among the children.

Variable	*N* = 97	%
*Age in years*		
2 to 3	31	32
4 to 5	31	32
6 and above	35	36
Mean (±SD) = 5.6 (±3.6)		
*Gender*		
Male	60	62
Female	37	38
*Family setup*		
Both parents	72	74
Single mother	23	24
Orphan/adopted	1	1
Abandoned	1	1
*Number of children in the family*		
1 child	55	57
2 children	25	26
3 children	14	14
4 children	3	3
*Siblings' history of intellectual disability*		
Yes	5	5
No	92	95
*Degree of intellectual disability (ID)*		
Mild ID	23	24
Moderate ID	39	40
Severe/profound ID	22	23
Unspecified ID	13	13

**Table 2:** Selected sociodemographic and economic characteristics of mothers.

Variable	*N* = 97	%
*Age of the mother*		
21–35 years	80	82.5
≥36 years	17	17.5
*Age of the mother at the time of child's birth*		
Below 20 years	6	6.2
21–35 years	84	86.6
≥36 years	7	7.2
*Age of the father*		
21–35 years	54	58.7
≥36 years	38	41.3
Missing	5	
*Age of the father at the time of birth of the child*		
21–35 years	68	73.9
≥36 years	24	26.1
Missing	5	
*Highest level of education of the mother*		
Primary level	31	32
Secondary level	56	57.7
College/university Level	10	10.3
*Highest level of education of the father*		
No formal education	2	2.2
Primary level	8	8.7
Secondary level	54	58.7
College/university Level	28	30.4
Missing	5	
*Occupation of the mother*		
Regular employment	30	30.9
Casual employment	46	47.4
Unemployed	21	21.6
*Occupation of the father*		
Regular employment	48	52.2
Casual employment	42	45.7
Unemployed	2	2.2
Missing	5	
*Whether the father smokes*		
Yes	27	29.3
No	65	70.7
Missing	5	
*Average monthly income in the family in Kenyan Shillings*		
More than 50,000	4	4.1
21,000 to 50,000	52	53.6
11,000 to 20,000	38	39.2
Less than 10,000	3	3.1

**Table 3:** Relationship between sociodemographic characteristics of children and severity of intellectual disability.

Variables	Severe/profound		Mild/moderate		OR	95% CI		*χ* ^2^ test
	*N*	%	*N*	%		Lower	Upper	*P* value
*Age in years*								
2 to 3	8	42.1%	11	57.9%	2.10	0.64	6.87	0.219
4 to 5	5	16.7%	25	83.3%	0.58	0.17	1.96	0.379
6 and above	9	25.7%	26	74.3%	1.00			
*Gender*								
Male	12	23.5%	39	76.5%	0.71	0.26	1.90	0.490
Female	10	30.3%	23	69.7%	1.00			
*Family setup*								
Both parents	15	23.4%	49	76.6%	1.00			
Single mother	7	38.9%	11	61.1%	2.08	0.69	6.31	0.196
*Number of children in the family*								
1 child	9	19.1%	38	80.9%	1.00			
2 children	6	28.6%	15	71.4%	1.69	0.51	5.57	0.389
3-4 children	7	43.8%	9	56.3%	3.28	0.96	11.19	0.057
*Siblings' history of intellectual disability*								
Yes	1	25.0%	3	75.0%	0.94	0.09	9.50	0.956
No	21	26.3%	59	73.8%	1.00			

OR = odds ratio; CI = confidence interval; *χ*^2^ = Chi-square.

**Table 4:** Association between sociodemographic and economic characteristics of mothers and severity of intellectual disability.

Variables	Severe/profound		Mild/moderate		OR	95% CI		*χ* ^2^ test
	*N*	%	*n*	%		Lower	Upper	*P* value
*Age of the mother*								
21–35 years	15	21.7%	54	78.3%	1.00			
≥36 years	7	46.7%	8	53.3%	3.15	0.98	10.09	0.053
*Age of the mother at the time of birth of the child*								
Below 20 years	2	33.3%	4	66.7%	1.25	0.12	13.24	0.853
21–35 years	18	25.4%	53	74.6%	0.85	0.15	4.76	0.852
>36 years	2	28.6%	5	71.4%				
*Age of the father*								
21–35 years	8	17.4%	38	82.6%	0.44	0.15	1.26	0.120
>36 years	11	32.4%	23	67.6%	1.00			
*Age of the father at the time of birth of the child*								
21–35 years	12	20.7%	46	79.3%	0.56	0.19	1.68	0.296
>36 years	7	31.8%	15	68.2%	1.00			
*Highest level of education of the mother*								
Primary level	8	30.8%	18	69.2%	0.74	0.14	3.88	0.722
Secondary level	11	22.0%	39	78.0%	0.47	0.10	2.28	0.349
College/university Level	3	37.5%	5	62.5%	1.00			
*Highest level of education of the father*								
Primary level	2	28.6%	5	71.4%	0.90	0.14	5.66	0.911
Secondary level	9	20.0%	36	80.0%	0.56	0.19	1.70	0.309
College/university Level	8	30.8%	18	69.2%	1.00			
*Occupation of the mother*								
Regular employment	8	30.8%	18	69.2%	1.00			
Casual employment	6	14.6%	35	85.4%	0.39	0.12	1.28	0.120
Unemployed	8	47.1%	9	52.9%	2.00	0.56	7.09	0.283
*Occupation of the father*								
Regular employment	13	30.2%	30	69.8%	2.60	0.82	8.20	0.096
Casual employment	5	14.3%	30	85.7%	1.00			
Unemployed								
*Whether the father smokes*								
Yes	3	12.0%	22	88.0%	0.33	0.09	1.27	0.096
No	16	29.1%	39	70.9%	1.00			
*Average monthly income in the family (in KES)*								
<21,000	8	23.5%	26	76.5%	0.79	0.29	2.16	0.647
21,000 and more	14	28.0%	36	72.0%	1.00			

OR = odds ratio; CI = confidence interval; *χ*^2^ = chi-square.

**Table 5:** Relationship between pregnancy-related factors and severity of intellectual disability.

Variables	Severe/profound		Mild/moderate		OR	95% CI		*χ* ^2^ test
	*n*	%	*N*	%		Lower	Upper	*P* value
*Attending ANC during pregnancy of the child*								
Yes	22	27.2%	59	72.8%	1.00			
No	0	0.0%	3	100.0%	UD	UD	UD	0.293
*Frequency of attending ANC *								
1-2 times	3	16.7%	15	83.3%	0.46	0.12	1.79	0.256
3-4 times	19	30.2%	44	69.8%	1.00			
*Took any drugs during pregnancy*								
Yes	11	47.8%	12	52.2%	4.17	1.46	11.87	**0.006**
No	11	18.0%	50	82.0%	1.00			
*Smoking during pregnancy*								
Yes	1	10.0%	9	90.0%	0.28	0.03	2.35	0.215
No	21	28.4%	53	71.6%	1.00			
*Living in environment where people smoke*								
Yes	12	18.2%	54	81.8%	0.18	0.06	0.55	**0.001**
No	10	55.6%	8	44.4%	1.00			
*Using alcohol during this pregnancy*								
Yes	5	35.7%	9	64.3%	1.73	0.51	5.88	0.375
No	17	24.3%	53	75.7%	1.00			

OR = odds ratio; CI = confidence interval; *χ*^2^ = chi-square; UD = undefined.

**Table 6:** Association between birth history of the children and severity of intellectual disability.

Variables	Severe/profound		Mild/moderate		OR	95% CI		*χ* ^2^ test
	*N*	%	*N*	%		Lower	Upper	*P* value
*Place of deliver for the baby*								
Health facility	22	28.6%	55	71.4%	1.00			
Home	0	0.0%	7	100.0%	UD	UD	UD	0.100
*Gestational age when the child born*								
From 33–37 weeks	7	31.8%	15	68.2%	1.43	0.49	4.17	0.510
Over 37 weeks	15	24.6%	46	75.4%	1.00			
*Labor complications*								
Yes	18	39.1%	28	60.9%	5.46	1.66	18.02	**0.003**
No	4	10.5%	34	89.5%	1.00			
*Mode of delivery*								
Spontaneous vaginal delivery	11	17.7%	51	82.3%	1.00			
Cesarean section	11	50.0%	11	50.0%	4.64	1.61	13.38	**0.005**
*Birth weight *								
<2.5 kg	5	27.8%	13	72.2%	1.11	0.34	3.57	0.863
2.5 Kg and above	17	25.8%	49	74.2%	1.00			
*Apgar score at birth*								
<7/10 (did not cry)	17	38.6%	27	61.4%	4.41	1.44	13.46	**0.007**
>7/10 (cried immediately)	5	12.5%	35	87.5%	1.00			
*Whether the baby was resuscitated at birth*								
Yes	16	40.0%	24	60.0%	4.22	1.45	12.29	**0.006**
No	6	13.6%	38	86.4%	1.00			

OR = odds ratio; CI = confidence interval; *χ*^2^ = chi-square; UD = undefined.

**Table 7:** Association between neonatal medical history of the children and severity of intellectual disability.

Variables	Severe/profound		Mild/moderate		OR	95% CI		*χ* ^2^ test
	*N*	%	*N*	%		Lower	Upper	*P* value
*Any neonatal difficulties*								
Yes	19	36.5%	33	63.5%	5.57	1.49	20.75	**0.006**
No	3	9.4%	29	90.6%	1.00			
*Whether the baby was admitted in NICU*								
Yes	18	41.9%	25	58.1%	6.66	2.01	22.03	**0.001**
No	4	9.8%	37	90.2%	1.00			
*Neonatal breathing difficulty*								
Yes	14	37.8%	23	62.2%	2.97	1.08	8.15	**0.031**
No	8	17.0%	39	83.0%	1.00			
*Neonatal seizures*								
Yes	6	30.0%	14	70.0%	1.29	0.42	3.91	0.657
No	16	25.0%	48	75.0%	1.00			
*Neonatal infection*								
Yes	4	20.0%	16	80.0%	0.64	0.19	2.17	0.471
No	18	28.1%	46	71.9%	1.00			
*Neonatal jaundice*								
Yes	3	27.3%	8	72.7%	1.07	0.26	4.44	0.930
No	19	26.0%	54	74.0%	1.00			
*Neonatal feeding difficulties*								
Yes	8	50.0%	8	50.0%	3.86	1.23	12.09	**0.016**
No	14	20.6%	54	79.4%	1.00			

OR = odds ratio; CI = confidence interval; *χ*^2^ = chi-square; UD = undefined.

**Table 8:** Association of infant and childhood medical history with severity of intellectual disability.

Variables	Severe/profound		Mild/moderate		OR	95% CI		*χ* ^2^ test
	*N*	%	*n*	%		Lower	Upper	*P* value
*Immunization history*								
Fully immunized	20	25.0%	60	75.0%	1.00			
Not fully immunized	2	50.0%	2	50.0%	3.00	0.40	22.71	0.287
*Suffer from any disease*								
Yes	10	22.7%	34	77.3%	0.69	0.26	1.82	0.449
No	12	30.0%	28	70.0%				
*History of meningitis*								
Yes	6	20.0%	24	80.0%	0.59	0.20	1.73	0.336
No	16	29.6%	38	70.4%				
*History of encephalitis*								
Yes	0	0.0%	6	100.0%	UD	UD	UD	0.130
No	22	28.2%	56	71.8%	1.00			
*History of cerebral malaria*								
Yes	2	66.7%	1	33.3%	6.10	0.53	70.90	0.104
No	20	24.7%	61	75.3%	1.00			
*History of head injury*								
Yes	2	18.2%	9	81.8%	0.59	0.12	2.96	0.571
No	20	27.4%	53	72.6%	1.00			
*History of severe malnutrition*								
Yes	3	23.1%	10	76.9%	0.82	0.20	3.31	0.781
No	19	26.8%	52	73.2%	1.00			
*Breastfeeding history*								
Breastfeed < 1 month	1	33.3%	2	66.7%	1.14	0.09	14.78	0.919
Breastfeed 1–24 months	13	22.8%	44	77.2%	0.68	0.23	1.99	0.477
Breastfeed > 24 months	7	30.4%	16	69.6%	1.00			

OR = odds ratio; CI = confidence interval; *χ*^2^ = chi-square.

**Table 9:** Relationship between pre-existing/co-morbid and severity of intellectual disability.

Variables	Severe/profound		Mild/moderate		OR	95% CI		*χ* ^2^ test
	*N*	%	*n*	%		Lower	Upper	*P* value
*Cerebral palsy*								
Yes	20	47.6%	22	52.4%	18.18	3.88	85.14	**≤0.001**
No	2	4.8%	40	95.2%	1.00			
*Convulsive disorders*								
Yes	19	27.5%	50	72.5%	1.52	0.39	5.99	0.547
No	3	20.0%	12	80.0%	1.00			
*Cardiovascular disease*								
Yes	1	50.0%	1	50.0%	2.91	0.17	48.53	0.438
No	21	25.6%	61	74.4%	1.00			
*Asthma*								
Yes	1	50.0%	1	50.0%	2.91	0.17	48.53	0.438
No	21	25.6%	61	74.4%	1.00			
*Pneumonia*								
Yes	6	26.1%	17	73.9%	0.99	0.33	2.96	0.989
No	16	26.2%	45	73.8%	1.00			
*Malnutrition*								
Yes	3	33.3%	6	66.7%	1.47	0.34	6.48	0.606
No	19	25.3%	56	74.7%				
*Rickets*								
Yes	1	25.0%	3	75.0%	0.94	0.09	9.50	0.956
No	21	26.3%	59	73.8%	1.00			
*Autism Spectrum Disorder (ASD)*								
Yes	1	10.0%	9	90.0%	0.28	0.03	2.35	0.215
No	21	28.4%	53	71.6%	1.00			
*Attention Deficit/Hyperactivity Disorder (ADHD)*								
Yes	2	22.2%	7	77.8%	0.79	0.15	4.10	0.774
No	20	26.7%	55	73.3%				

OR = odds ratio; CI = confidence interval; *χ*^2^ = chi-square.

**Table 10:** Factors associated with severity of intellectual disability among children.

Variable	COR	95% CI		*P* value	AOR	95% CI		*P* value
		Lower	Upper			Lower	Upper	
*Labor complications*								
Yes	5.46	1.66	18.02	0.003	9.45	1.23	113.29	**0.036**
No	1.00				1.00			
*Mode of delivery*								
Spontaneous vaginal delivery	1.00				1.00			
Cesarean section	4.64	1.61	13.38	0.005	3.48	0.59	20.56	0.169
*APGAR score at birth*								
<7/10	4.41	1.44	13.46	0.007	9.15	0.47	179.54	0.145
>7/10	1.00				1.00			
*Whether the baby was resuscitated at birth*								
Yes	4.22	1.45	12.29	0.006	0.08	0.00	3.37	0.186
No	1.00				1.00			
*Any neonatal difficulties/complications at birth*								
Yes	5.57	1.49	20.75	0.006	2.13	0.10	46.61	0.631
No	1.00				1.00			
*Whether the baby was admitted in NICU*								
Yes	6.66	2.01	22.03	0.001	8.09	2.11	31.07	**0.002**
No	1.00				1.00			
*Neonatal breathing difficulty*								
Yes	2.97	1.08	8.15	0.031	0.26	0.02	2.92	0.272
No	1.00				1.00			
*Neonatal feeding difficulties*								
Yes	3.86	1.23	12.09	0.016	0.52	0.09	3.23	0.486
No	1.00				1.00			
*Cerebral palsy*								
Yes	18.18	3.88	85.14	0.001	21.18	4.18	107.40	**≤0.001**
No	1.00				1.00			
*Took any drugs during pregnancy*								
Yes	4.17	1.46	11.87	0.006	3.92	0.70	21.97	0.120
No	1.00				1.00			
*Living in environment where people smoke*								
Yes	0.18	0.06	0.55	0.001	0.09	0.11	1.31	0.092
No	1.00				1.00			

COR = crude odds ratio; CI = confidence interval; AOR = adjusted odds ratio.
